# Testosterone increases the virulence traits of uropathogenic *Escherichia coli*

**DOI:** 10.3389/fmicb.2024.1422747

**Published:** 2024-05-28

**Authors:** Rongrong Wu, Carolina Pettersson, Isak Demirel

**Affiliations:** ^1^School of Medical Sciences, Örebro University, Örebro, Sweden; ^2^Department of Clinical Research Laboratory, Faculty of Medicine and Health, Örebro University, Örebro, Sweden

**Keywords:** uropathogenic *Escherichia coli*, testosterone, growth, virulence, cross-kingdom interaction

## Abstract

Uropathogenic *Escherichia coli* (UPEC) is the most common cause of urinary tract infections (UTIs) in humans. Testosterone negatively impacts UTIs by affecting the immune response, leading to higher susceptibility of chronic cystitis in individuals with elevated testosterone levels, regardless of gender. Current research is mostly focused on how testosterone affects the host response to UPEC, but not so much is known about how testosterone directly affect UPEC virulence. The aim of the present study was to investigate the impact of testosterone exposure on the virulence of UPEC. We found that testosterone directly increases UPEC growth, endotoxin release and biofilm formation. We also found that testosterone-stimulated CFT073 increased colonization and invasion of bladder epithelial cells. Testosterone-stimulated CFT073 also increased the release of IL-1β and LDH from bladder epithelial cells. Additionally, by using a *Caenorhabditis elegans* survival assay we also showed that testosterone decreased the survival of CFT073 infected *C. elegans* worms. Taken together, our findings show that testosterone directly increases the virulence traits of UPEC.

## 1 Introduction

Urinary tract infections (UTIs) represent one of the most prevalent infectious diseases in women. Uropathogenic *Escherichia coli* (UPEC) is the most common cause of UTIs, accounting for over 80% of cases. Over 50% of all women will experience a UTI at some point in their lives (Flores-Mireles et al., 2015; Terlizzi et al., 2017). Additionally, young children, individuals with diabetes, immunocompromised individuals, and patients with catheters are also at a high risk of developing UTIs and recurrent UTIs. UTIs can range from asymptomatic to cystitis, pyelonephritis, and even urosepsis (Tamadonfar et al., 2019). Approximately 1% of cystitis patients progress to pyelonephritis, and an even smaller percentage advance to sepsis. After recovery, nearly 25% of UTI patients experience a recurrent UTI within six months, and 45% have a recurrence within one year (Flores-Mireles et al., 2015; Klein and Hultgren, 2020). More than half of these recurrences are caused by the same UPEC isolate that caused the initial infection (Klein and Hultgren, 2020).

Testosterone appears to have a detrimental effect on UTIs by influencing the immune response in both males and females. Individuals with higher levels of testosterone, regardless of gender, are more prone to developing chronic cystitis (Deltourbe et al., 2022). Studies indicate that female mice treated with testosterone or dihydrotestosterone before a UPEC infection struggled to clear the infection, whereas untreated female mice typically resolved their infection within four weeks. Moreover, mouse strains with elevated testosterone levels, such as male C3H/HeN mice, exhibited increased susceptibility to chronic cystitis (Deltourbe et al., 2022). Castration of male mice improved their ability to resolve the infection, while reintroducing testosterone resulted in similar bacterial burden compared to the untreated males (Deltourbe et al., 2022). These findings strongly suggest that testosterone contributes to the severity and persistence of UTIs. In addition, after menopause, the ratio of estrogen to testosterone is reduced in women (less estrogen, more stable testosterone levels), which might also affect the outcome of UTIs (Villa et al., 2012).

Presently, much of the research in the field of host-pathogen interaction focuses on understanding how pathogens, armed with their virulence factors, manipulate or circumvent immune responses to initiate infections. However, there’s a notable gap in the knowledge concerning the influence of host immune factors, such as cytokines and sex hormones, on the virulence of UPEC through cross-kingdom interactions. Most current research on UTIs in relation to the sex hormones testosterone and estrogen, has primarily concentrated on their impact on the host and the analysis of risk factors. It has been demonstrated by Wang et al. (2013) that mice with diminished estrogen levels exhibit higher bacteriuria levels compared to controls. Moreover, elevated testosterone levels in women have been linked to chronic cystitis (Deltourbe et al., 2022). It has been shown that cytokines can bind to bacterial DNA and modify gene expression in *Neisseria meningitidis* (Mahdavi et al., 2013). We have also shown that pro-inflammatory cytokines can alter UPEC virulence, resulting in a reduction of *Caenorhabditis elegans* (*C. elegans*) survival (Engelsöy et al., 2019). In addition, the effect of estradiol on *Pseudomonas aeruginosa* leads to an enhancement of its virulent mucoid biofilm phenotype (Chotirmall et al., 2012). We have recently demonstrated that post-menopausal concentrations of estradiol, but not pre-menopausal concentrations, significantly increased the growth and biofilm formation of UPEC. Additionally, we discovered that the pre-menopausal concentration of estradiol significantly decreased UPEC-mediated death of *C. elegans* (Engelsoy et al., 2021). These studies reinforce the notion that estrogen directly influences bacterial virulence. However, little is known about how testosterone affects the virulence of UPEC and whether this could explain the clinical association between testosterone levels and the outcome of UPEC-mediated UTI. The aim of this study was to investigate the impact of testosterone exposure on the virulence of UPEC.

## 2 Materials and methods

### 2.1 Bacteria culture

CFT073 is a fully sequenced UPEC strain isolated from a patient with pyelonephritis (Mobley et al., 1990). CFT073 containing an GFP expression pLMB499 plasmid (a kind gift from Prof. Philip Poole from University of Oxford, United Kingdom) was used for colonization experiments. CFT073 deletion mutants CFT073Δ*fimH*, CFT073Δ*pap*, and CFT073Δ*hlyA* were created using λ Red Recombinase (Demirel et al., 2018). All bacteria were maintained on tryptic soy agar plates (Becton Dickinson, Franklin Lakes, NJ, United States).

### 2.2 Growth assessment

CFT073 was grown in Lysogeny broth (Becton Dickinson, Franklin Lakes, NJ, United States) overnight on a shaker at 37°C before the growth assay. 1 × 10^6^ CFU/mL CFT073 was grown in minimal salt medium [MSM contains 0.3% KH_2_PO_4_, 1.3% (wt/vol) Na_2_HPO_4_, 0.05% NaCl, 0.1% NH_4_Cl, 20 mM glucose, 2 mM MgSO_4_, 100 mM CaCl_2_, and 0.25% casamino acids] with or without the presence of testosterone (100 pg/mL–60 ng/mL, Merck, Darmstadt, Germany) in a 96-well-plate. DMSO was used as a vehicle control. The optical density (600 nm) was measured every 10 min for 24 h using the spectrophotometer Cytation 3 (Biotek Inc., Winooski, VT, United States) at 37°C.

### 2.3 Biofilm formation

Following the growth assay, the same 96-well plate was utilized for assessing biofilm formation after a period of 24 h. The wells were wash with sterile RO water three times. A 0.1% crystal violet solution (Thermo Fisher Scientific, Waltham, MA, United States) was used for biofilm staining. Any remaining crystal violet was washed away with sterile RO water three times, and the plate was left to air dry overnight. Subsequently, 95% ethanol was used to dissolve the crystal violet, which was then transferred to a new plate. The absorbance (540 nm) was quantified using a spectrophotometer (Cytation 3).

### 2.4 LAL assay

CFT073 was grown in MSM with or without testosterone (100 pg/mL–60 ng/mL) statically at 37°C for 24 h. DMSO was used as a vehicle control. The bacteria were centrifuged 5,000 g for 5 min, and the supernatants were sterile filtered through a 0.2 μm filter and stored at −80°C. The Pierce LAL chromogenic endotoxin quantitation kit (Thermo Fisher Scientific) was used for endotoxin quantification according to the manufacturer’s protocol.

### 2.5 Hemolysis assay

Human erythrocytes were isolated from healthy blood donors and purified with density gradient centrifugation as previously described (Lindblad et al., 2022). An ethical approval has been granted by the regional ethics review board in Uppsala, Sweden (Dnr 2015/437), to isolate blood from healthy individuals after informed consent. Blood from healthy donors were collected according to the ethical guidelines of both the Declaration of Helsinki and the Swedish national board of health and welfare. Hemolysis was evaluated by stimulating erythrocytes (40 million cells/ml, diluted in MSM) with 5 × 10^8^ CFU/mL CFT073 for 4–8 h. CFT073 was cultured in advance with or without testosterone in MSM statically for 24 h at 37°C. DMSO was used as a vehicle control. After centrifugation, hemoglobin leakage was evaluated at 404 nm using a spectrophotometer (Cytation 3).

### 2.6 Cell culture

Human bladder epithelial cell line 5,637 was purchased from American Type Culture Collection (Manassas, VA, United States) and cultured with Dulbecco’s Modified Eagle Medium (DMEM) (Lonza, Basel, Switzerland) supplemented with 10% fetal bovine serum (FBS), 2 mM L-glutamine and 1 mM non-essential amino acids (all from Thermo Fisher Scientific) at 37°C with 5% CO_2_. The cell experiments were carried out within 10 passages. Before infection experiments, the cell medium was changed to DMEM with charcoal filtered FBS for 30 h to minimize hormonal effects. The cells were shifted to DMEM with charcoal filtered FBS for 30 h to minimize hormonal effects.

### 2.7 Colonization assay

CFT073 that contains an enhanced GFP-expression plasmid (eGFP), was primed with testosterone (100 pg/mL–60 ng/mL) in MSM and grown statically for 24 h at 37°C. DMSO was used as a vehicle control. Culturing was followed by 2 times wash with MSM to remove free testosterone. Bladder epithelial cell line 5,637 was seeded in a 96-well-plate (5 × 10^4^ cells/well) and infected with CFT073 at multiplicity of infection (MOI) 10 for 4 h. After infection, the cells were washed 10 times with PBS, as previously described (Lindblad et al., 2019). Adhered/invaded (referred to as colonized) CFT073 (eGFP) were quantified and imaged with the Cytation 3 plate reader. Colonization is presented as mean fluorescence intensity (MFI) after subtraction of background fluorescence.

### 2.8 Invasion assay

In brief, the CFT073 was grown in MSM with or without testosterone (100 pg/mL–60 ng/mL) statically at 37°C for 24 h, followed by washing 2 times with MSM to remove free testosterone. DMSO was used as a vehicle control. The bladder epithelial cell line 5,637 was seeded in a 24-well-plate (2.5 × 10^4^ cells/well), and infected with CFT073 at MOI 100 for 2 h, followed by PBS wash 10 times. The remaining extracellular bacteria was killed by adding 100 μg/mL gentamicin for 2 h. The cells were washed with PBS 10 times and lysed with 0.1% Triton X-100 (v/v PBS containing MgCl_2_ and CaCl_2_) for 10 min under rotation at 300 rpm. The lysates were plated on TSA plates for overnight incubation, and the colonies were counted next day.

### 2.9 ELISA and LDH release

CFT073 was grown in MSM with or without testosterone (100 pg/mL–60 ng/mL) statically at 37°C for 24 h, followed by washing 2 times with MSM to remove free testosterone. DMSO was used as a vehicle control. The bladder epithelial cell line 5,637 was seeded in a 24-well-plate (2.5 × 10^4^ cells/well) and infected with CFT073 at MOI 10 for 4 h. The supernatants were collected and centrifuged at 5,000 g for 5 min and stored at −80°C for further experiments. CyQUANT LDH assay (Thermo Fisher Scientific) was used to measure the LDH release according to manufacturer’s protocol.

Beta-defensin 1 (NBP2-67933, Bio-Techne, Minneapolis, MN, United States), Beta-defensin 2 (NBP2-77363, Bio-Techne), LL37 (NBP3-06932, Bio-Techne), RNase7 (ab215418, Abcam, Cambridge, United Kingdom), IL-1β and IL-8 (ELISA MAX Deluxe Sets, BioLegend, San Diego, United States) were analysed by enzyme-linked immunosorbent assay (ELISA) according to the manufacturer’s instructions.

### 2.10 *Caenorhabditis elegans* toxicity assay

The *Caenorhabditis elegans* (*C. elegans*) wild-type Bristol strain N2 (Caenorhabditis Genetics Centre, University of Minnesota, United States) was maintained on nematode growth plate lawned with *E. coli* OP50 (Caenorhabditis Genetics Centre) at 21°C. Prior to the experiment, the nematodes were synchronized to L4 stage. After washing, around 20 worms were added to each well and stimulated with 5 × 10^8^ CFU/mL CFT073 in the presence or absence of testosterone for 12 h at 21°C. DMSO was used as a vehicle control. The viability of the worms was evaluated every hour. A worm was counted dead when it failed to respond to touch.

### 2.11 Statistics

All the statistic were performed with Prism 9.0. Student’s unpaired *t*-test or one-way ANOVA followed by Dunnett multiple testing. The data were presented with mean + standard deviation (SD). Results were considered statistically significant at *p* < 0.05. *n* is equal to the number of independent biological experiments.

## 3 Results

### 3.1 Testosterone promotes increased UPEC growth

**Figure 1:**
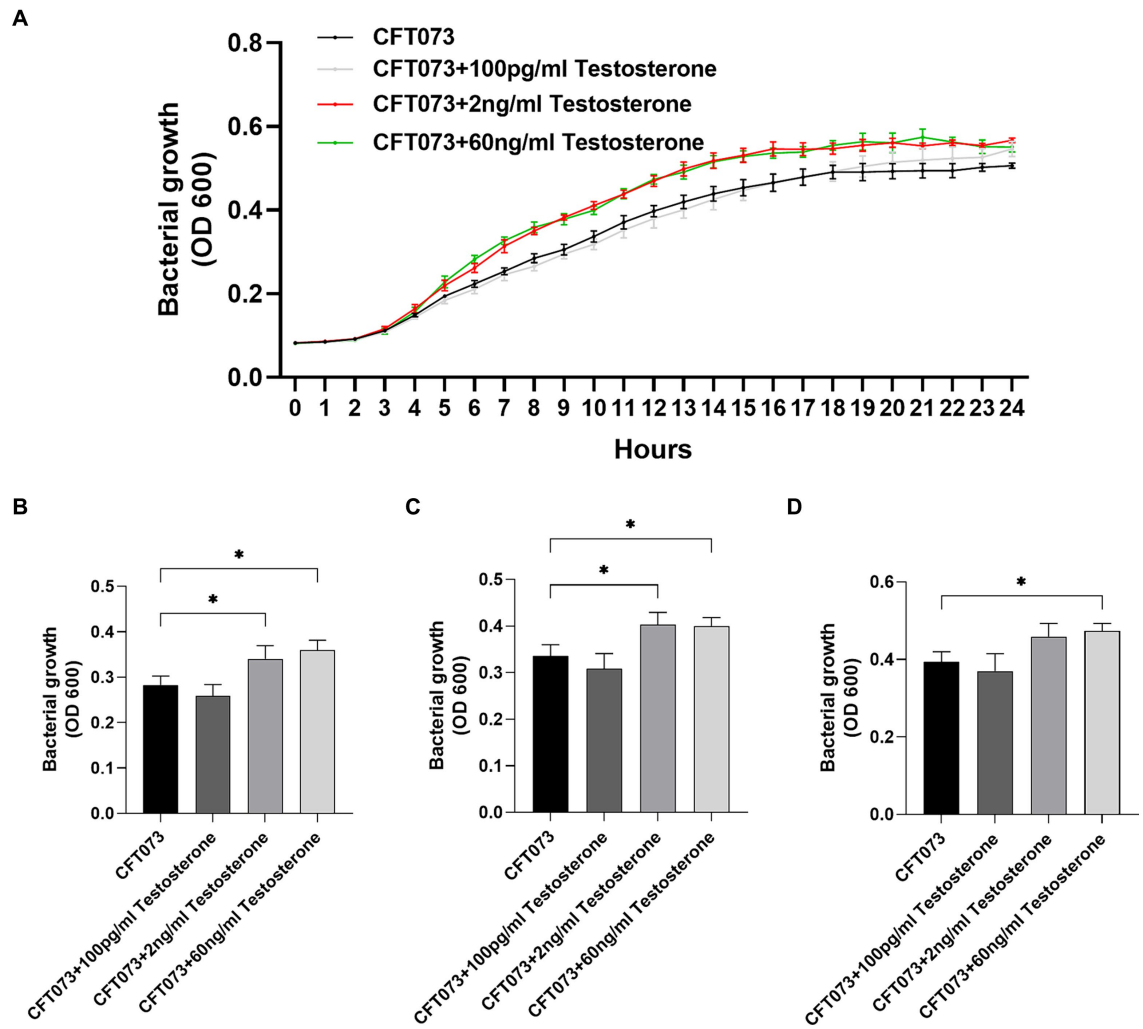
CFT073 growth with or without the presence of testosterone (100 pg/mL, 2 ng/mL and 60 ng/mL) during 24 h **(A)** and at 8 **(B)**, 10 **(C)** and 12 **(D)** hours. Data are presented as mean **(A)** and mean ± SD **(B)** of *n* = 3 independent experiments. The asterisk distinguishes statistical significance: * = *p* < 0.05 vs. CFT073.

To investigate the potential biological effects of testosterone on CFT073, we began with evaluating the growth of CFT073 with or without the presence of testosterone. We found that 2 ng/mL and 60 ng/mL testosterone, but not 100 pg/mL of testosterone significantly increased the bacterial growth after 8 h (Figure 1B), 10 h (Figure 1C) and 12 h (Figure 1D) compared to unstimulated CFT073 (Figures 1A–D). In addition, we also found that 2–40 ng/mL of testosterone significantly induced increased CFT073 growth compared to unstimulated CFT073 (Supplementary Figures S1A–D).

### 3.2 Testosterone increased biofilm formation and endotoxin release

**Figure 2:**
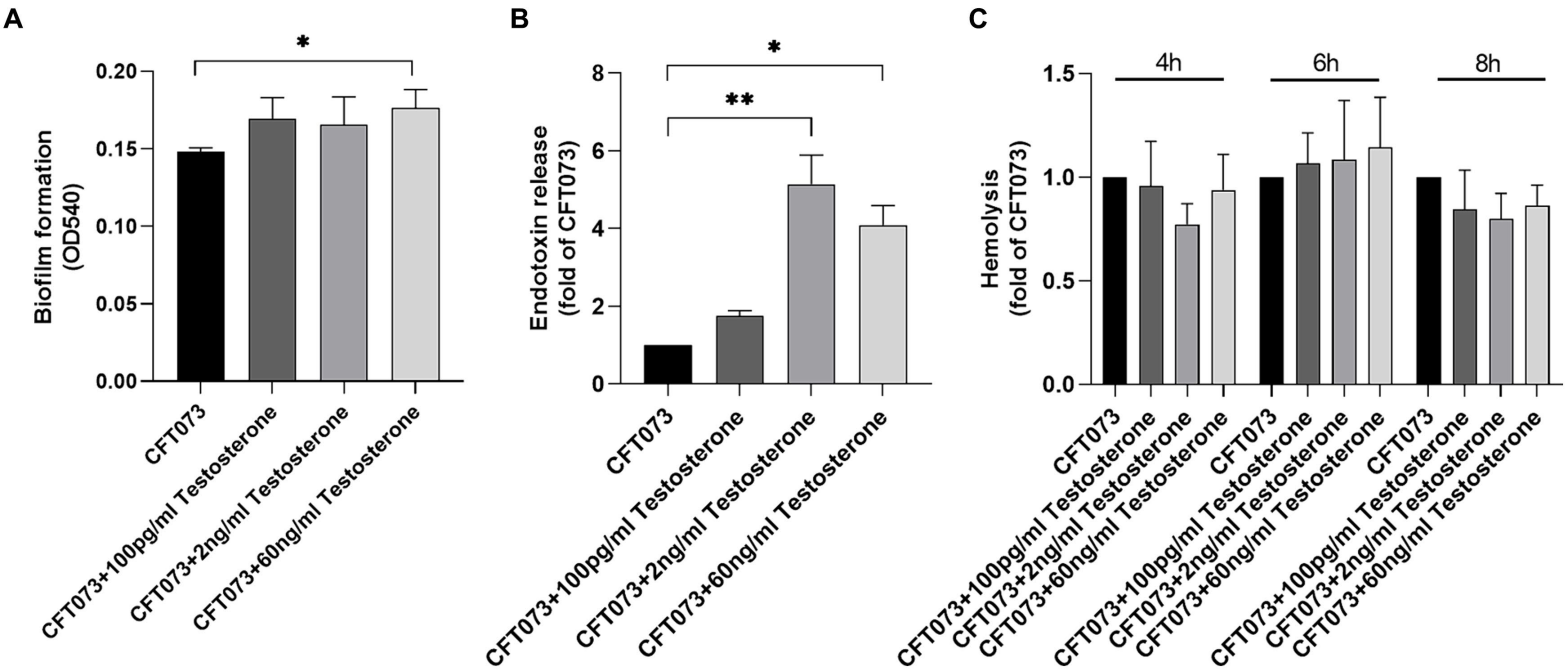
Biofilm formation **(A)**, endotoxin release **(B)** and hemolysis **(C)** with or without the presence of testosterone (100 pg/mL, 2 ng/mL and 60 ng/mL) after 4–8 **(C)** or 24 h **(A–B)**. Data are presented as mean ± SD of *n* = 3–5 independent experiments. The asterisk distinguishes statistical significance: * = *p* < 0.05 and ** = *p* < 0.01 vs. CFT073.

We proceeded with evaluating the effects of testosterone on key virulence traits of UPEC. First, we evaluated the effects of testosterone on biofilm formation. We found that only 60 ng/mL of testosterone significantly increased biofilm formation of CFT073 compared to unstimulated CFT073 after 24 h (Figure 2A; Supplementary Figure S1E). We also found that 2 ng/mL and 60 ng/mL, but not 100 pg/mL of testosterone significantly increased endotoxin release from CFT073 compared to unstimulated CFT073 (Figure 2B). However, we could not observe any testosterone mediated changes to CFT073s hemolysis capacity compared to unstimulated CFT073 after 4–8 h (Figure 2C).

### 3.3 Testosterone promotes increased colonization and invasion of bladder epithelial cells

**Figure 3:**
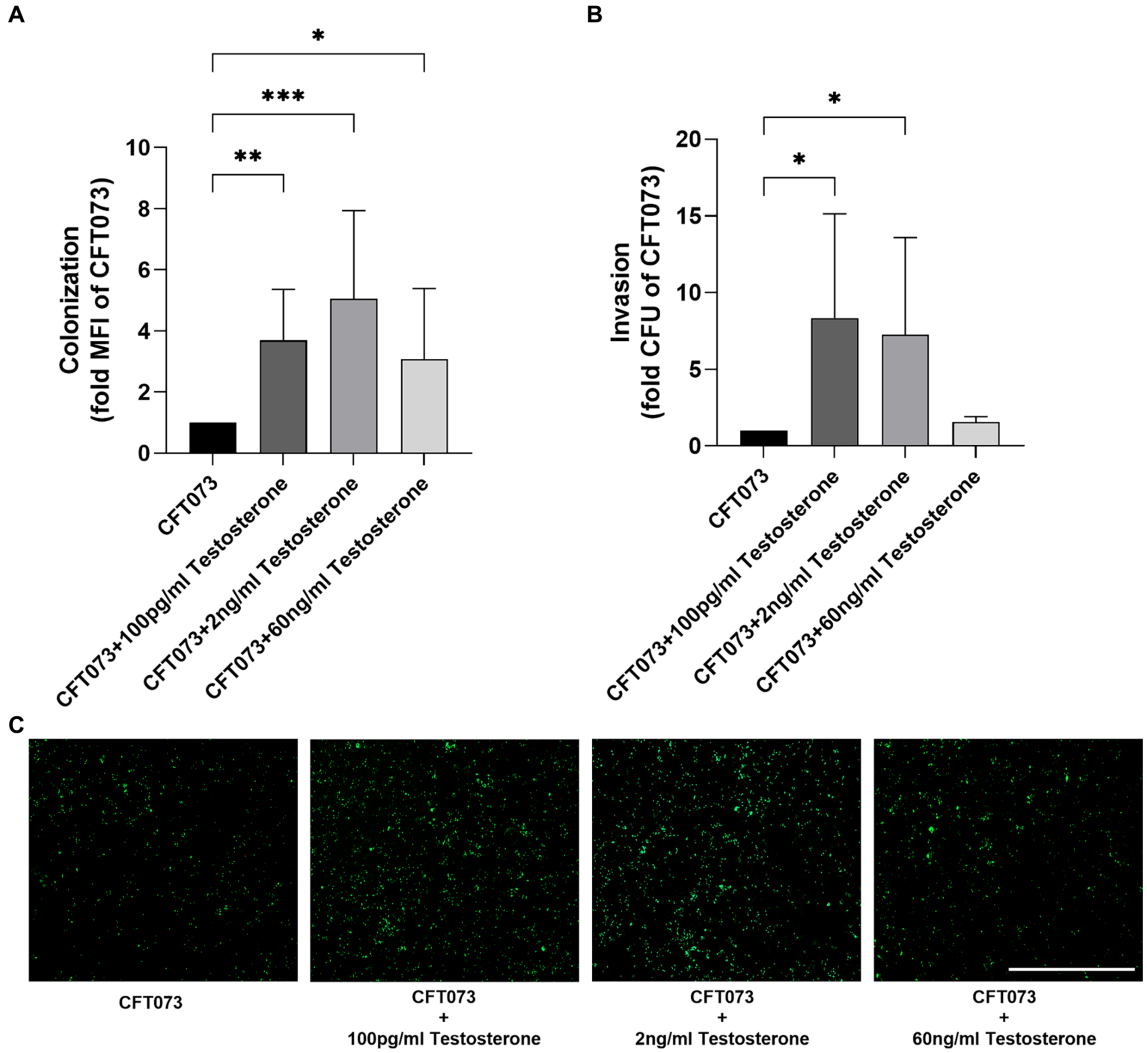
CFT073 colonization (**A,C** 4 h) or invasion (**B**, 2 h) of bladder epithelial cells after CFT073 pre-treated with or without testosterone (100 pg/mL, 2 ng/mL and 60 ng/mL). CFT073 (carrying a GFP-expressing plasmid) colonization was measured as mean fluorescence intensity (MFI) **(A)** and imaged **(C)**. Data are presented as mean ± SD of *n* = 3–8 independent experiments. The asterisk distinguishes statistical significance: * = *p* < 0.05, ** = *p* < 0.01, and *** = *p* < 0.001 vs. CFT073. Scale bar: 500 μm.

We continued with investigating the effects of testosterone on UPECs ability to colonize (adhere and invade) and invade human bladder epithelial cells. We found that 100 pg/mL, 2 ng/mL and 60 ng/mL of testosterone significantly increased colonization compared to unstimulated CFT073 (Figures 3A,C). We also found that 100 pg/mL and 2 ng/mL, but not 60 ng/mL of testosterone significantly increased the invasion capacity of CFT073 compared to unstimulated CFT073 (Figure 3B).

### 3.4 Testosterone alters cytokine and LDH release, but not antimicrobial peptides

**Figure 4:**
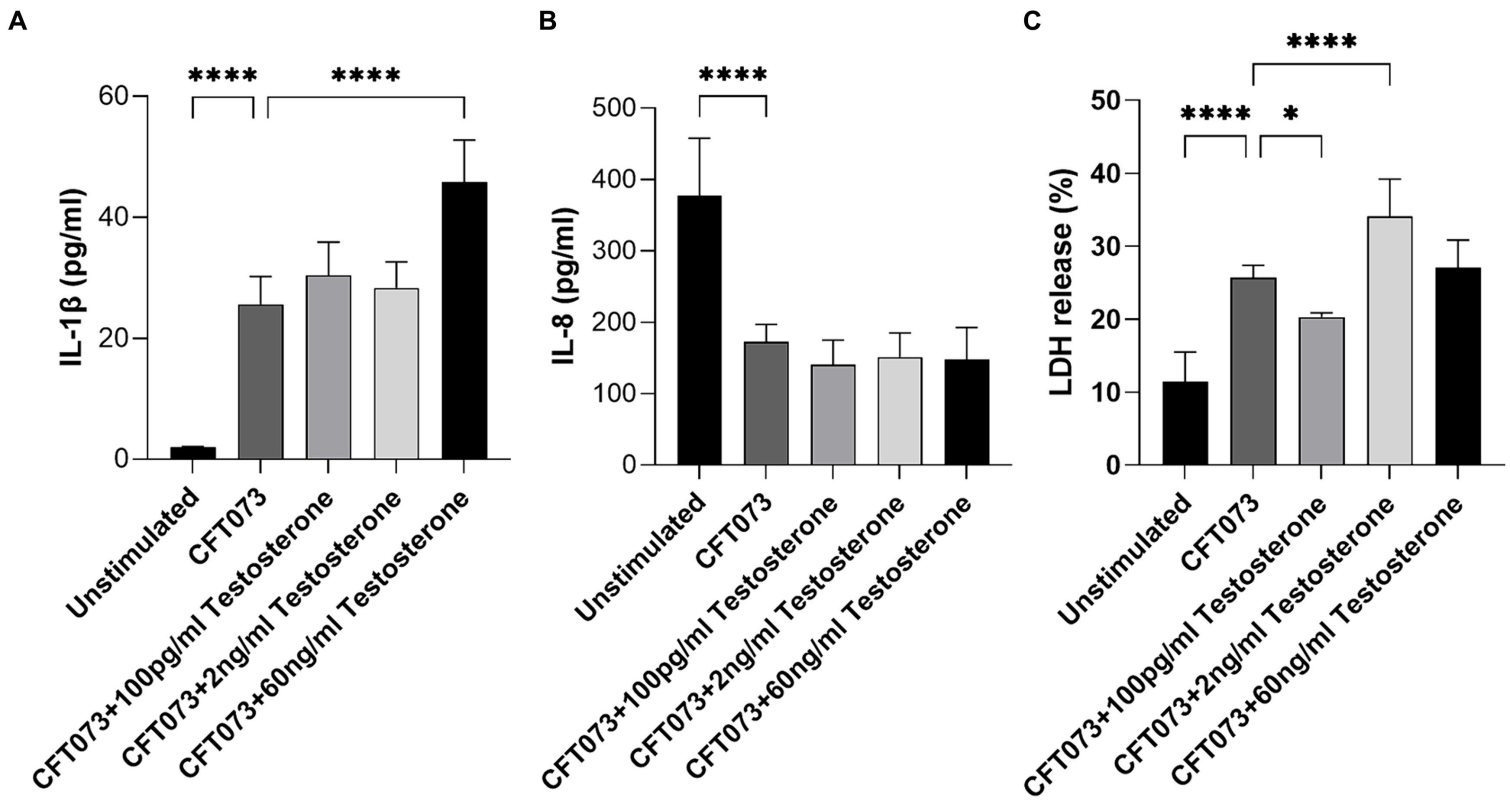
IL-1β **(A)**, IL-8 **(B)** and LDH **(C)** release from bladder epithelial cells stimulated with CFT073 MOI10 pre-treated with or without testosterone (100 pg/mL, 2 ng/mL and 60 ng/mL) at 4 h. Data are presented as mean ± SD of *n* = 6–8 independent experiments. The asterisks distinguish statistical significance: * = *p* < 0.05 and **** = *p* < 0.0001.

Next, we evaluated if testosterone-stimulated CFT073 could alter the release of IL-1β, IL-8 and LDH from bladder epithelial cells. Intriguingly, IL-1β (Figure 4A), but not IL-8 (Figure 4B), release was significantly increased upon infection with 60 ng/mL testosterone-stimulated CFT073 compared to unstimulated CFT073. We also found that IL-8 release was significantly inhibited by CFT073 alone (Figure 4B), and that IL-1β release was significantly increased by CFT073 alone (Figure 4A) compared to unstimulated cells. Furthermore, we found that 2 ng/mL was the only concentration of testosterone-stimulated CFT073 that significantly increased the LDH release from bladder epithelial cells compared to unstimulated CFT073 (Figure 4C). In contrast, we found that 100 pg/mL of testosterone-stimulated CFT073 significantly reduced LDH release compared to CFT073 alone (Figure 4C).

**Figure 5:**
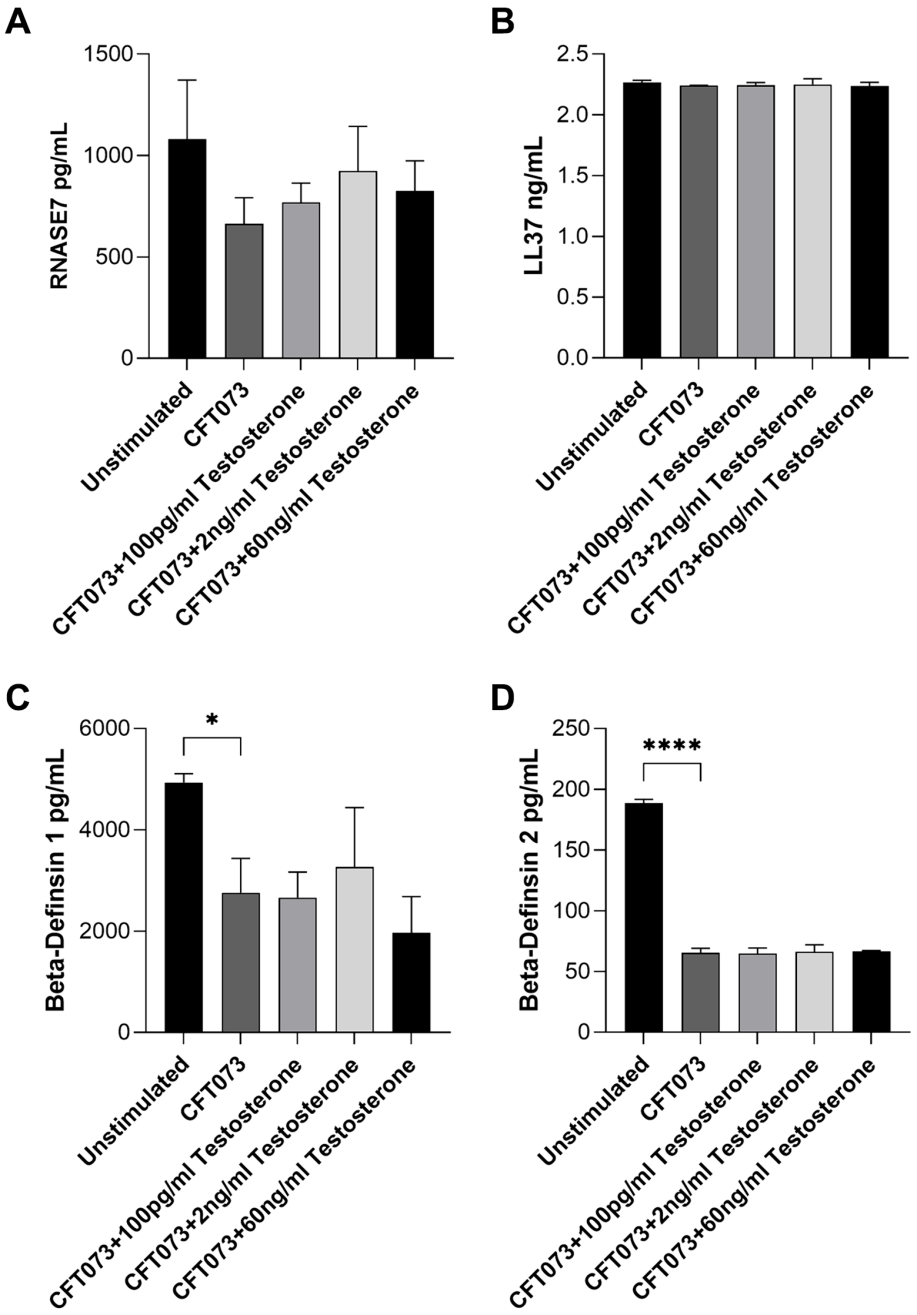
RNASE7 **(A)**, LL37 **(B)**, beta-defensin 1 **(C)** and beta-defensin 2 **(D)** release from bladder epithelial cells stimulated with CFT073 MOI10 pre-treated with or without testosterone (100 pg/mL, 2 ng/mL and 60 ng/mL) at 4 h. Data are presented as mean ± SD of *n* = 3 independent experiments. The asterisks distinguish statistical significance: * = *p* < 0.05 and **** = *p* < 0.0001.

We also evaluated the effects of testosterone-stimulated CFT073 on antimicrobial peptide release from bladder epithelial cells. We found that testosterone-stimulated CFT073 did not alter the release of RNASE7, LL-37, beta-defensin 1 or beta-defensin 2 compared to unstimulated CFT073 (Figure 4). However, we did find that beta-defensin 1 (Figure 5C) and beta-defensin 2 (Figure 5D) release was significantly reduced by CFT073 alone, while RNASE7 (Figure 5A) and LL-37 (Figure 5B) showed no significant reduction compared to unstimulated cells.

### 3.5 Testosterone increased CFT073 toxicity to *Caenorhabditis elegans*

**Figure 6:**
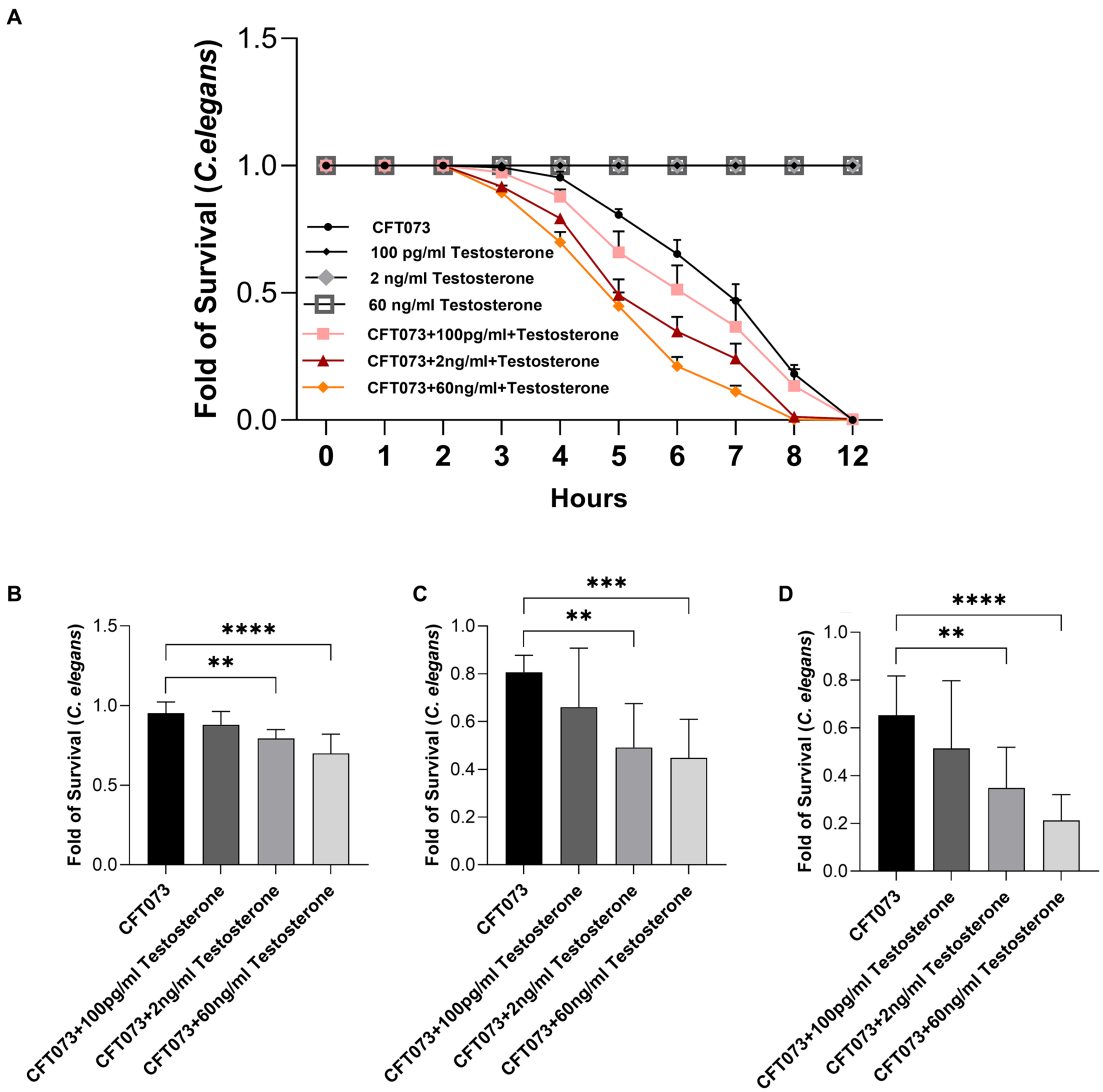
*Caenorhabditis elegans* survival after stimulation with CFT073 pre-treated with or without testosterone (100 pg/mL, 2 ng/mL and 60 ng/mL) for 4 **(B)**, 5 **(C)**, 6 **(D)** and 12 h **(A)**. Data are presented as mean ± SD of *n* = 9 independent experiment. Statistical significance is denoted with asterisks: ** = *p* < 0.01, *** = *p* < 0.001, and **** = *p* < 0.0001 vs. CFT073.

A *C. elegans* infection model was utilized to investigate general toxicity of CFT073 in the presence or absence of testosterone. We found that CFT073 in the presence of 2 ng/mL and 60 ng/mL testosterone significantly decreased *C. elegans’* survival compared to unstimulated CFT073 (Figure 6A). We observed the significantly reduced *C. elegans* survival after 5 h (Figure 6B), 6 h (Figure 6C) and 7 h (Figure 6D). Testosterone *per se* did not induce any *C. elegans* toxicity (Figure 6A).

## 4 Discussion

Testosterone is detrimental for UTIs because it appears to alter the immune response in both male and female. Males, as well as females with higher levels of testosterone, are more likely to develop chronic cystitis (Deltourbe et al., 2022). Studies have shown that female mice treated with testosterone or dihydrotestosterone before UPEC infection showed an inability to eliminate the infection, whereas untreated female mice naturally resolved their infection within 4 weeks. Furthermore, mouse strains with higher testosterone levels, such as male C3H/HeN mice, were more susceptible to chronic cystitis (Deltourbe et al., 2022). Androgen exposure also impaired neutrophil function and potentiated invasion of tubular cells during a UPEC infection (Olson et al., 2018; Hreha et al., 2024). Hence, we know that there is a clinical association between testosterone levels, and the development of UPEC-mediated UTIs. However, we do not know if the direct effects of testosterone on UPEC virulence could partially explain the association between testosterone levels and UPEC-mediated UTI. In this study we focused on investigating the cross-kingdom effects of testosterone on the virulence of UPEC.

Our findings showed that 2 and 60 ng/mL of testosterone significantly increased the growth of the UPEC strain CFT073. We and others have previously shown that the sex hormone estradiol can increase the growth of several gram-negative bacteria (Mahdavi et al., 2013; Wang et al., 2013; Engelsoy et al., 2021), but we found that this growth increase was associated only with post-menopausal concentrations of estradiol. However, we have not found any prior references to testosterone inducing increased *E. coli* growth. Yet, testosterone was shown to increase *Actinobacillus seminist* growth (Ramírez-Paz-Y-Puente et al., 2023). As bacterial growth is strongly associated with UTIs (Anderson et al., 2004), these findings may be a contributing factor explaining why women are more prone to UTI after menopause. After menopause, the ratio of estrogen to testosterone is reduced in women, which also might have a net effect on UPEC virulence and the outcome of a UTI (Villa et al., 2012).

We also found that biofilm formation was increased by testosterone. Others have also shown that sex hormones can increase the biofilm formation of gram-negative bacteria (Wang et al., 2013; Fteita et al., 2014). Biofilm formation is linked to protection of UPEC from environmental conditions, antimicrobial agents and the host immune responses (Mah and O’Toole, 2001; Hatt and Rather, 2008). We also investigated if testosterone could alter the hemolytic capacity of UPEC and the release of endotoxin. We found that both 2 and 60 ng/mL of testosterone increased endotoxin release from UPEC, but not UPECs hemolytic capacity. It is known that UPEC LPS activates and modulates the host immune response during a UTI (Bussolati et al., 2002; Blomgran et al., 2004). Taken together, we have shown that testosterone induces increased bacterial growth, biofilm formation and endotoxin release, which may promote UPEC persistence in the urinary tract.

UPECs capacity to adhere and invade bladder epithelial cells is essential for the colonization of the urinary tract (Anderson et al., 2003; Justice et al., 2004). We observed an increased UPEC colonization and invasion of bladder epithelial cells mediated by testosterone. If we factor in testosterones effects on bladder epithelium, which promotes colonization and persistence (Deltourbe et al., 2022), it makes these findings even stronger. Bladder epithelial cells express β1-integrins and uroplakins, which interact with UPEC type-1 fimbriae. This interaction is important for CFT073 adhesion and invasion into bladder epithelial cells (Eto et al., 2007). Taken together, these findings show that testosterone promotes better UPEC colonization of bladder epithelial cells, which is strongly associated with increased virulence.

We proceeded to investigate if CFT073 pre-stimulated with testosterone could alter the cytokine, chemokine and LDH release from bladder epithelial cells. We showed that IL-1β but not IL-8, release was increased upon infection with testosterone-stimulated CFT073. We also found that testosterone-stimulated CFT073 increased the LDH release from bladder epithelial cells. The observed increased IL-1β and LDH release may be linked, as pyroptosis (LDH release and caspase-1 activation) is associated with IL-1β release from bladder epithelial cells (Nagamatsu et al., 2015; Demirel et al., 2018). Furthermore, it has been shown that UPEC a-hemolysin induced caspase-1/4-dependent cell death in bladder epithelial cells, which was associated with the increased IL-1b release (Nagamatsu et al., 2015). It has also been shown that IL-1β release from mice infected with UPEC contributes to the severity of the infection. Mice that were lacking IL-1β were shown to be protected from developing UTIs (Ambite et al., 2016). Hence, our findings support the hypothesis that testosterone increases the virulence of UPEC. IL-8 is also a very important cytokine during a UTI. IL-8 is important for neutrophil recruitment, which is the primary immune cell that clears the infection (Hedges and Svanborg, 1994; Godaly et al., 1997). To summarize, we have shown that testosterone-stimulated CFT073 increased IL-1β and LDH release from bladder epithelial cells, which may aggravate the infection.

The body’s initial defense against UPEC involves the release of antimicrobial peptides (White et al., 2022). These peptides exhibit potent immunomodulatory properties, which include triggering the production of cytokines, chemokines, and tight junction proteins (Niyonsaba et al., 2009; Akiyama et al., 2014). Our findings showed that UPEC could suppress the release of several antimicrobial peptides and that testosterone did not alter this ability. Hence, CFT073s maximum suppression capacity of antimicrobial peptide release may have been reached at this MOI. In the future it would be interesting to evaluate a lower concentration of CFT073. This to elucidate if the testosterone-stimulated CFT073 could potentially enhance UPECs ability to suppress antimicrobial peptide release.

An *in vivo C. elegans* infection model was used to investigate general toxicity of CFT073 in the presence or absence of testosterone. It has been demonstrated that there is a correlation between the virulence of UPEC observed in a mouse model and in *C. elegans* (Diard et al., 2007; Engelsöy et al., 2019; Engelsoy et al., 2021). We showed that CFT073 in the presence of 2 ng/mL and 60 ng/mL testosterone significantly decreased *C. elegans*’ survival. We hypothesise that the decreased survival could be due to the increased bacterial growth and the increased endotoxin release. *E. coli* LPS has been shown to induce reduced *C. elegans* survival in a dose-dependent way (Ma et al., 2020). However, several additional UPEC virulence factor like the vacuolating autotransporter toxin (Vat) and secreted autotransporter toxin (Sat), that we have not evaluated may contribute to the decreased survival. Both virulence factors have been shown to mediate tissue damage (Wiles et al., 2008; Engelsöy et al., 2019). We have previously also shown that estradiol-stimulated CFT073 mediated less *C. elegans* toxicity (Engelsoy et al., 2021). Strengthening the hypothesis that estradiol reduces, and testosterone increases the virulence of UPEC. To summarize, we have shown that testosterone, increases the total cytotoxicity of CFT073.

In the current era of escalating antibiotic resistance worldwide, gaining insights into how UPEC interacts with and alters the human host for urinary tract colonization is becoming increasingly crucial. There is evidence to suggest that human factors, such as cytokines and hormones, which are produced and released by our cells, can trigger the virulence of various bacteria (Mahdavi et al., 2013). If we can gain a deeper understanding of how UPEC perceives its surroundings and mobilizes its virulence, we might be able to suppress this activation, thereby mitigating or completely preventing the infection. By focusing on inhibition of virulence, we decrease the antibiotic selection pressure, which could lead to reduced antibiotic resistance (Brannon and Hadjifrangiskou, 2016). We have shown that testosterone increases the virulence of UPEC by mediating increased growth, endotoxin release, biofilm formation, colonization, cytokine and LDH release and *C. elegans* toxicity. As the ratio of estrogen to testosterone is reduced in women after menopause (Villa et al., 2012), our study suggests that all these factors may be contributing factors to why women are more prone to UTI after menopause. Although we have found that testosterone can alter the virulence of UPEC, additional research is needed to clarify the mechanism behind these findings and what clinical significance they may have.

## Data availability statement

The original contributions presented in the study are included in the article/Supplementary material, further inquiries can be directed to the corresponding author.

## Ethics statement

The studies involving humans were approved by regional ethics review board in Uppsala, Sweden (Dnr 2015/437). The studies were conducted in accordance with the local legislation and institutional requirements. The participants provided their written informed consent to participate in this study. The manuscript presents research on animals that do not require ethical approval for their study.

## Author contributions

RW: Conceptualization, Data curation, Formal analysis, Investigation, Methodology, Writing – original draft, Writing – review & editing. CP: Conceptualization, Data curation, Formal analysis, Investigation, Methodology, Writing – review & editing. ID: Conceptualization, Data curation, Formal analysis, Funding acquisition, Investigation, Methodology, Supervision, Writing – original draft, Writing – review & editing.
