# Supplementary material for: Testosterone increases the virulence traits of uropathogenic Escherichia coli
Source: Front Microbiol. 2024 May 28;15:1422747. doi: 10.3389/fmicb.2024.1422747 (PMC11165178; doi:10.3389/fmicb.2024.1422747)
Supplement: Supplementary file 1 [file Data_Sheet_1.docx]

Supplementary Material


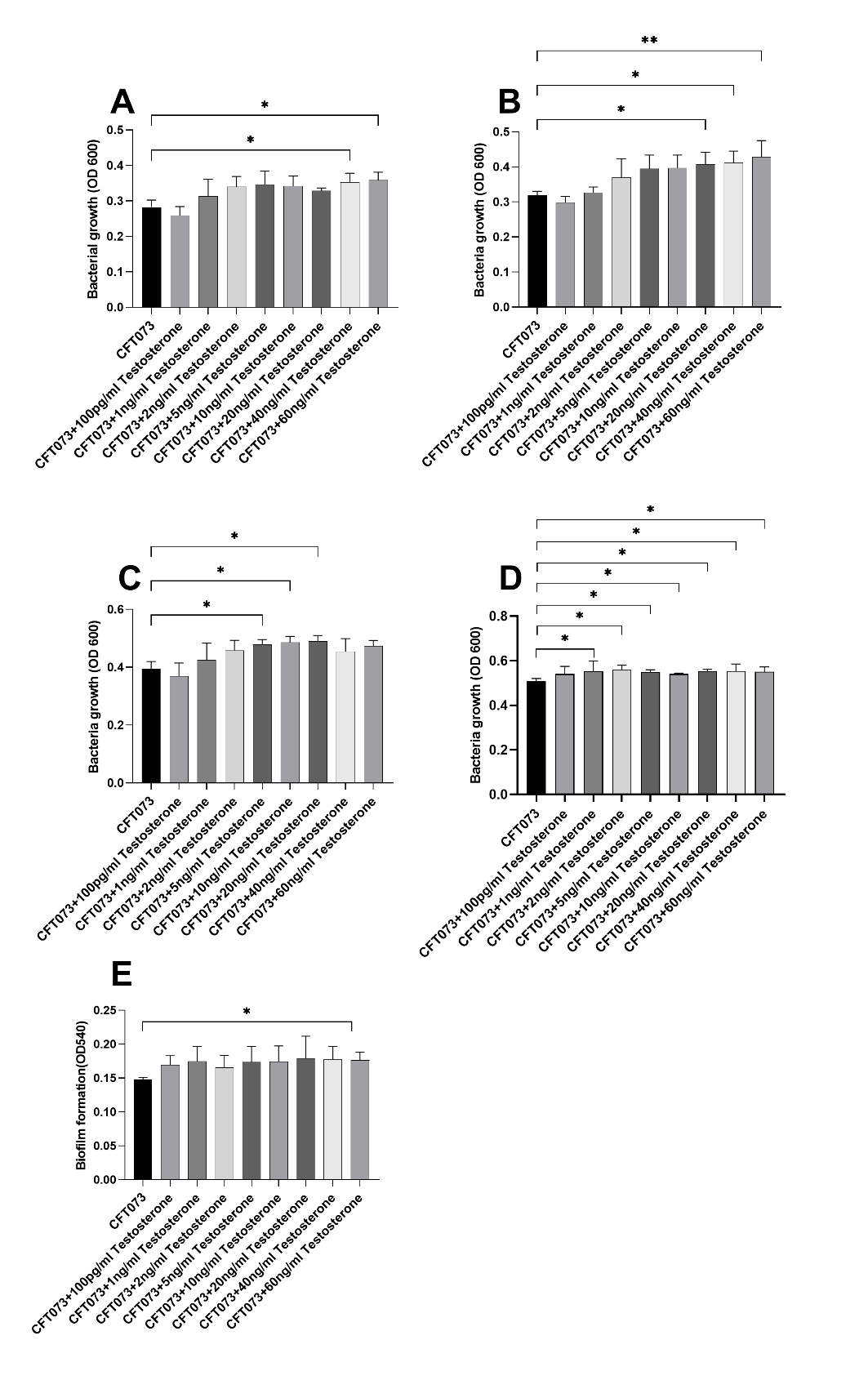


**Supplementary Figure S1.** CFT073 growth or biofilm formation with or without the presence of testosterone (100pg/ml-60 ng/ml) during 8 (A) and at 10 (B), 12 (C) and 24 (D-E) hours. Data are presented as mean ± SD of n=3-5 independent experiments. The asterisk distinguishes statistical significance: *=p<0.05, *=p<0.01 vs. CFT073.
